# The increasing utilization of ventricular assist devices in fontan failure

**DOI:** 10.1016/j.jhlto.2025.100282

**Published:** 2025-05-29

**Authors:** Darren Turner, Amir Mehdizadeh-Shrifi, Grant Chappell, David L. Morales

**Affiliations:** The Heart Institute, Cincinnati Children’s Hospital Medical Center, University of Cincinnati College of Medicine, Cincinnati, OH

**Keywords:** Fontan, Fontan-associated heart failure, Ventricular assist devices, Single Ventricle, Heart Failure, Mechanical Circulatory Support, Cardiac transplantation

## Abstract

The Fontan population has increased dramatically owing to advances in medical and surgical therapies, with many living well into adulthood with Fontan circulation. Unfortunately, patients will develop heart failure due to the chronic effects of their altered circulatory system. Management of heart failure in these patients is very complex and requires multi-disciplinary approaches with input from both cardiologists and surgeons. In the case of patients who develop cardiogenic shock, transplantation is often not feasible due to instability. Recently, there has been increased use of ventricular assist devices (VADs) as a bridge to transplantation with promising results. In this work, we briefly review the physiology of Fontan failure, provide criteria for VAD workup, and discuss VAD outcomes in Fontan patients. Finally, we describe a single institution’s experience and outcomes with VADs in Fontan patients.

## Introduction

The Fontan procedure is the final stage in single-ventricle palliation. Since its introduction in 1971, significant advancements in preoperative, perioperative, and postoperative care have greatly improved survival.^1^ Notably, transplant-free survival rates have been reported as high as 94% at 10 years and 87% at 20 years.^2^ The success of the Fontan procedure has led to a growing population of pediatric patients with Fontan circulation, with an estimated 70,000 individuals worldwide.^3^ Unfortunately, these patients’ unique circulatory systems subject them to the risk of long-term complications, such as chronic venous hypertension, systolic dysfunction, diastolic dysfunction, and reduced cardiac output. While medical therapies can offer temporary symptom relief, there is a lack of strong evidence supporting their long-term effectiveness, resulting in considerable variability in treatment approaches across institutions.^4^, ^5^, ^6^ About one-third of Fontan patients will require heart transplantation within 35 years of Fontan surgery.^7^ Coupled with the fact that we continue to have a shortage of available donor organs, this has led to an increased use of mechanical circulatory support (MCS).^8^ In this review, we aim to explore the pathophysiology of Fontan failure, the considerations for ventricular assist device (VAD) workup, and discuss VAD outcomes as a bridge to transplantation in Fontan patients.

## Fontan physiology and circulation

**Figure 1:**
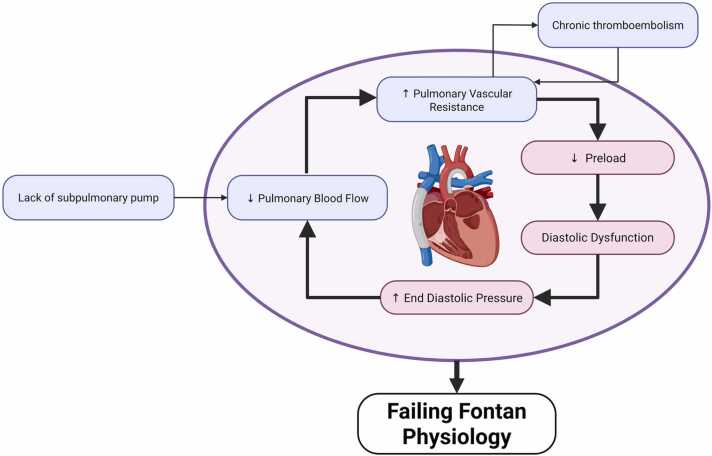
Fontan pathophysiology cycle.

The classic Fontan circulation has features including low cardiac output, altered ventricular function, chronic venous hypertension, leading to multi-organ dysfunction, which may progress to failure (Figure 1). Other notable downstream end-organ complications of Fontan circulation include plastic bronchitis (PB), Fontan-associated liver dysfunction (FALD), renal dysfunction, and protein-losing enteropathy (PLE).^9^, ^10^, ^11^, ^12^, ^13^, ^14^, ^15^ These sequelae adversely affect transplant outcomes.^16^, ^17^

Contributing to the low cardiac output state is the passive forward flow mechanics in the venous system, which decreases systemic ventricle preload. Indeed, compared to adult cases of heart failure, the effect of inotropes in certain Fontan patients is not as efficacious because of the limited preload.^18^ This decreased pre-load over time leads to the chronic underfilling of the systemic ventricle and the development of diastolic dysfunction, which leads to increased systemic filling pressures that can further inhibit the passive pulmonary flow of the Fontan circulation. This progressive unloading of the systemic ventricle, coined as ‘disuse hypofunction’ by Gewillig et al, refers to the resultant remodeling in Fontan circulation from longstanding volume deprivation. The vicious cycle of disuse hypofunction, loss of subpulmonary pulsatility, cyanosis, contributes to exercise intolerance in patients with Fontan circulation.^3^, ^19^

Arrhythmias, atrial or ventricular, can develop after the Fontan operation. This could be due to a number of factors, including surgical manipulation of the atria, ventriculotomy, scar tissue formation, concomitant atrioventricular valve replacement, or increased atrial pressures, to name a few.^20^, ^21^ These arrhythmias can lead to impairment of both systolic and diastolic function.

Increased pulmonary vascular resistance is likely multifactorial in nature, but the lack of pulsatility and chronic hypoxia, which accompany Fontan physiology, are likely major contributors.^22^ The lack of pulsatility also limits the recruitable pulmonary blood flow and cardiac output, further contributing to diastolic dysfunction. One approach to improve filling and decrease venous pressure is to fenestrate the Fontan, potentially allowing for increased cardiac output. However, the cost of the fenestration is a decrease in systemic oxygen saturation due to increased right-to-left shunting.^23^

The pulmonary circulation is altered in several other ways. Staged palliation and systemic-to-pulmonary shunt further contribute to altered pulmonary blood flow, which results in a predisposition for acquired pulmonary hypoplasia and pulmonary vascular disease. The chronic venous hypertension discussed is further complicated by aortopulmonary and veno-venous collateral circulation. Aortopulmonary collaterals developed in Fontan circulation can constitute 11–62% of systemic cardiac output.^24^ In the setting of significant aortopulmonary collaterals, one must plan for VAD therapy to support a cardiac output 1.5 to > 2 times what would be expected for a patient of that size. Anecdotally, we have observed some resolution of collateral flow after a year of SVAD support, evidenced by having to decrease RPMs over time in the clinic in our chronically supported patients, decreasing flow from over 8 L to less than 6 L. Moreover, chronic venous hypertension leads to lymphatic overload with obstruction and the development of liver fibrosis, typical characteristics of FALD.^25^ It is also well documented that the lymphatic system in single ventricle patients can be extremely abnormal, leading to chronic ascites and pleural effusions.^26^

Finally, due to the low-flow state of the venous system that lacks a subpulmonary pump, there is an increased risk of thromboembolism.^27^ This risk of thromboembolism is highest within 1 year of surgery. However, the risk from chronic subclinical thromboembolism remains elevated for several years thereafter and may contribute to reduced pulmonary flow and progressive Fontan circulation failure.^28^

## Fontan VAD preoperative evaluation

The timing of Fontan failure is variable and depends on anatomy and the systemic ventricular morphology. Historically, Fontan anatomy was previously thought to be extremely challenging for mechanical circulatory support (MCS), but several congenital heart programs demonstrated successful use of MCS as a bridge to transplant^29^, ^30^, ^31^, ^32^, ^33^. It would become clear that the success of MCS therapy is predicated upon understanding each patient's unique physiologic and anatomic challenges.

The preoperative evaluation and timely referral for MCS intervention are probably the most significant factors in improving survival on durable VAD, as is in patients without CHD^34^. Clinical evaluation is multifaceted and includes cardiopulmonary, liver, renal, and hematologic consultation for preoperative optimization (Figure 1). Common complicating factors in these patients are malnutrition, sarcopenia, end-organ dysfunction, steroid use, and anticoagulation management.

### Cardiopulmonary workup

**Figure 2:**
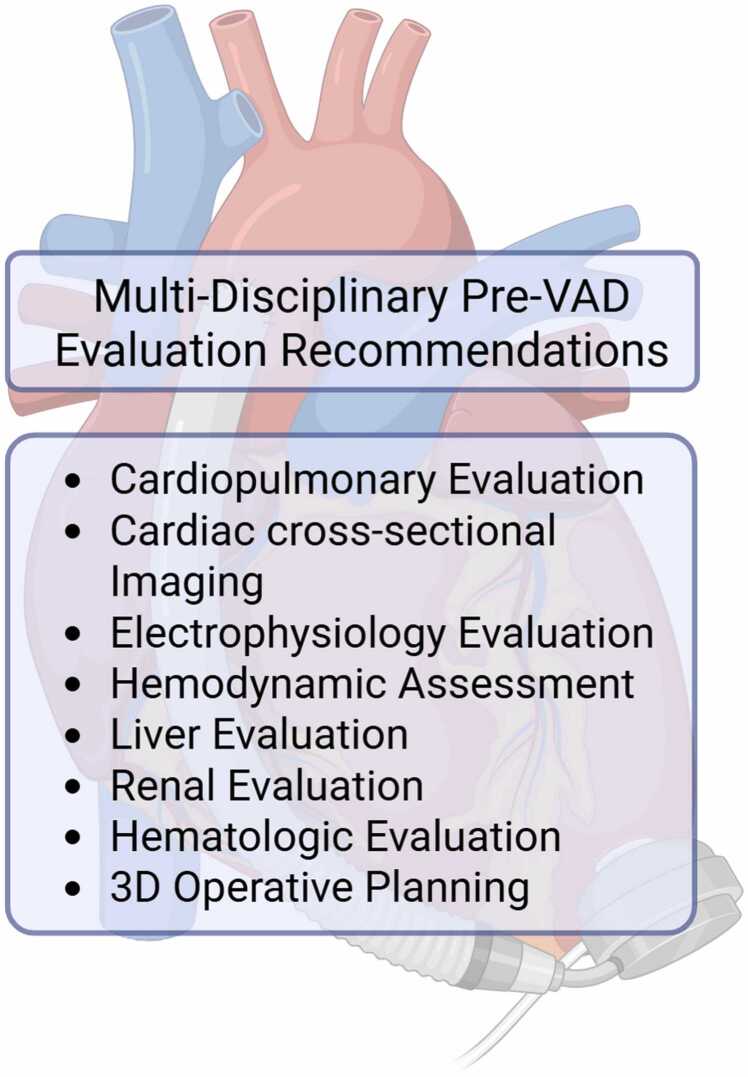
Fontan pre-VAD evaluation.

Cardiac workup should include cross-sectional imaging and electrophysiologic evaluation (Figure 2). Furthermore, patients with extensive aortopulmonary or veno-venous collaterals may require preoperative catheter intervention to close large vessels. Veno-venous collaterals should be evaluated for potential closure given the risk of significant postoperative cyanosis. However, this should be weighed against the benefits of Fontan fenestration, as it provides a passive source of VAD filling in cases where the patient requires positive pressure ventilation or experiences increased pulmonary vascular resistance. Cavopulmonary obstruction, pulmonary vascular disease, or obstruction of the common atrial connection also complicates the application of systemic VAD use due to the likelihood of inadequate filling. These issues must be addressed at the time of the VAD implantation. Finally, significant risk factors such as arrhythmias, atrioventricular valve regurgitation, and systemic right ventricles should be considered with surgical planning, as all three are associated with poorer outcomes.^35^, ^36^, ^37^, ^38^

Approximately two-thirds of patients with Fontan circulation demonstrate a mildly restrictive lung disease and a mild-to-moderate limitation in diffusion capacity on pulmonary function tests.^39^ Risk factors for this include multiple sternotomies, diaphragmatic paralysis, and scoliosis. Pulmonary function tests can aid risk stratification and preoperative optimization. The development and burden of plastic bronchitis (PB) is a significant manifestation of Fontan circulatory failure. Of course, corticosteroids, nebulized mucolytic agents, and cough therapy are a mainstay of pharmacological treatment, but no individual medical treatment has been shown to consistently treat PB.^40^ In refractory cases or significant development of bronchial casts, bronchoscopic interventions may be warranted.^41^ A work-up to assess the burden of plastic bronchitis may help optimize postoperative outcomes. PB can resolve after a period of excellent cardiac output and low venous pressures with VAD support.^42^ However, it does not always resolve and a clear understanding in whom it is reversible with VAD support is not known.

Hemodynamic assessment is crucial to operative planning and prognostication of VAD therapy. Fontan circulatory failure can occur due to either systemic ventricular failure, which tends to cause ‘classic’ heart failure symptoms such as exercise intolerance, whereas failure of the subpulmonary circulation portends effects on the lymphatic and venous systems, contributing to PLE, PB, and liver disease. Because of these different phenotypes, it was theorized that tailoring MCS to support the specific etiology of circulatory failure would improve outcomes. However, the reality is that patients usually present with a mix of both phenotypes, which may explain the mixed outcomes. For example, patients with diastolic systemic ventricular failure and subpulmonary circulatory failure will likely not benefit from only subpulmonary MCS, since increasing flow into an already restrictive ventricle will increase atrial pressures. Heart catheterization can deduce the etiology of circulation failure by assessing the end-diastolic pressure of the single ventricle. For those with pressures <12 mmHg and moderate or minimal dysfunction, placing a systemic VAD probably will not be beneficial. A limited fluid challenge test should be cautiously performed at the time of catheterization to truly understand the restrictive nature of the systemic ventricle.

Finally, preoperative optimization of cardiogenic shock is imperative for the best outcomes. At our institution, patients presenting in cardiogenic shock are often placed on temporary support such as an Impella® or Centrimag®. Once the patient has been resuscitated and the end-organ function normalized, we will move forward with durable VAD support. For those whose course is complicated by cardiac arrest, we recommend ECPR peripherally but usually move to central ECMO cannulation within 24 h. A systemic ventricle not working well and with the amount of collateral flow that exists in Fontan patients will often lead to significant atrial hypertension that cannot be adequately decompressed by a fenestration. Therefore, we would move to central ECMO with a cannula decompressing the common atrium. However, if the patient is large enough, we would use an Impella® to decompress the systemic side and continue with peripheral ECMO.

### Liver evaluation

Recently, it has been recognized that advanced and end-stage liver disease is a long-term complication of hepatic congestion caused by venous hypertension and the lack of a subpulmonary pump in Fontan circulation. Early-stage liver disease should be assessed as it often progresses without noticeable symptoms, prompting greater reliance on imaging techniques like computed tomography, ultrasound, and elastography for monitoring.^43^ However, using radiographic findings alone to assess the extent of liver damage has its limitations, and there should be a low threshold for performing a biopsy. It is important to note that even a biopsy may not always accurately reflect fibrosis in Fontan patients due to the patchy nature of the disease.^44^ The MELD-XI score has also been used in conjunction with liver biopsy as a model to estimate the extent of liver disease.^45^ In patients with end-stage liver disease, a combined heart-liver transplant has become a more common treatment approach.^46^ Another argument for the use of heart-liver transplants is the reduced rates of allograft rejection and longer graft survival.^46^, ^47^ The authors have been in favor of the en-bloc heart-liver transplants for Fontan patients as well as the use of VADs to bridge patients to this combined transplant.^48^ However, there are no standardized guidelines for combined heart-liver transplantation in Fontan patients. Because of the lack of guidelines, there remain questions about organ stewardship and how to best select candidates for heart-liver transplant as not to waste this precious resource.

### Kidney evaluation

The kidneys receive around 20–25% of cardiac output, and hemodynamic changes associated with Fontan circulation can significantly affect renal function. These effects include a decreased glomerular filtration rate and reduced renal perfusion.^49^, ^50^ Preoperative renal dysfunction has been linked to adverse outcomes following ventricular assist device (VAD) implantation ^51^ Kidney evaluation should include glomerular filtration rate, creatinine, and non-creatinine based tests. For example, cystatin-C-based testing can be used preoperatively and offers greater sensitivity than traditional creatinine-based measures, likely due to the sarcopenic characteristics of these patients. Given the impact of renal function on perioperative mortality, appropriate renal risk stratification should be conducted^52^, ^53^. However, one can often place a VAD, though with a higher risk, in those with poor renal function for the purpose of resuscitating the kidneys.

### Hematologic evaluation

The reported incidence of thromboembolism after Fontan surgery is between 17% and 33%.^54^, ^55^ Presumably, factors such as arrhythmias, increased pulmonary vascular resistance, and lack of pulsatile pulmonary blood flow contribute. There is also evidence that liver dysfunction and PLE contribute to an altered coagulation profile.^56^, ^57^ Several studies demonstrate an altered amount of coagulation factors, but these studies were not standardized or conducted in age-matched cohorts.^58^, ^59^ Still, there is considerable ongoing debate concerning an optimal anticoagulation regimen. A recent prospective, multicenter study by McCrindle was unable to find a statistically significant difference in the safety profile between rivaroxaban and ASA as anticoagulation in Fontan patients.^60^ Careful risk assessment and management are crucial to reduce thromboembolism-associated morbidity and mortality. Finally, it is worth mentioning that iron should be supplemented to maintain functional status. Because these patients are often cyanotic, they tend to have increased hemoglobin, hematocrit, and red blood cells. Because of this, iron stores are relatively low and are correlated with lower exercise capacity.^61^

### Operative modeling and precautions

**Figure 3:**
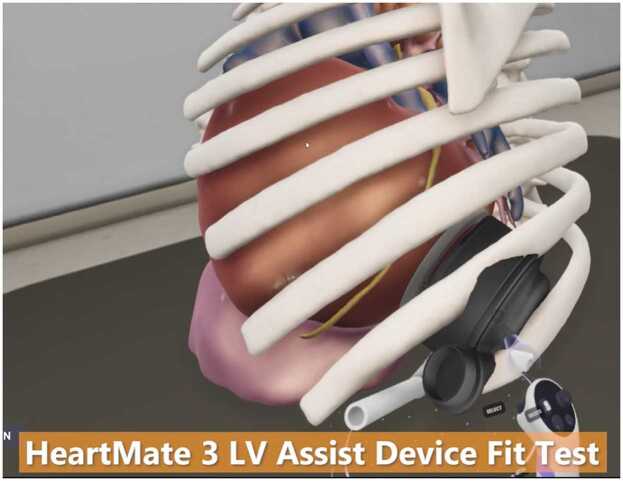
Pre-operative 3D modeling.

Over the past couple of decades, the application of three-dimensional modeling in patient care has grown exponentially. Given the unique nature of any CHD patient’s anatomy, it is not surprising that cross-sectional imaging used in combination with virtual reality-based systems has been applied to congenital heart surgery to help with surgical planning, particularly concerning pump position, position of atrioventricular valves, and great vessels.^62^, ^63^ A similar approach has been taken with the total artificial heart. Though more applicable to children and young adults, the aid of virtual planning has allowed for more concrete decision-making and better spatial recognition to understand anatomic limitations and size constraints (Figure 3).

Frailty was recently identified in 33% of Fontan patients was not readily identified by many providers.^64^ It cannot be stressed enough that many patients will be frail and sarcopenic at the time of evaluation. Multiple surgeries, exercise intolerance, PLE, and social factors may all contribute to frailty. Moreover, studies have demonstrated frailty to be an important predictor of adverse outcomes.^65^, ^66^ This phenomenon may be amenable to cardiopulmonary rehabilitation and counseling before and especially after VAD placement.^67^

## Mechanical support bridge to transplant outcomes

While numerous case reports have explored the use of ventricular assist devices (VADs) in Fontan patients, only recently have multicenter studies documented the outcomes following VAD implantation in failing Fontan circulation (Table 1). Cedars and colleagues conducted a retrospective analysis of the ACTION database, which examined systemic VAD use in pediatric patients with Fontan anatomy.^68^ Their study included 45 patients, 69% of whom had right ventricular morphology, and 83% demonstrated signs of systemic ventricular dysfunction. The results showed that 11 patients died while on support, 30 patients (67%) received heart transplants, and 3 patients remained on device support. The study reported a 1-year survival rate of 78%. Another retrospective study by Bedzra et al included a slightly larger cohort of 55 Fontan patients, which also included adults with congenital heart disease (CHD).^69^ Most patients receiving only systemic ventricular support had left ventricular morphology, although the authors noted that some RVADs might have been recorded as LVADs. This study reported a 6-month survival rate of 76%. Notably, both studies found worse outcomes for patients bridged to VAD support with ECMO. The largest study thus far is by Chen et. al and contained 106 total patients, with around 20% of patients being 18 years of age or older.^70^ In this study, they noted a mortality rate of 14% at 12 months for those who were under the age of 18% and 33% for those 18 years of age or older, highlighting the need for additional study of the adult population to minimize morbidity and mortality. As with the previous studies, most patients were INTERMACS 1 or 2 at VAD implantation, unfortunately. There should be a significant effort to avoid placing durable VADs in patients in INTERMACS 1. The authors would suggest implanting a temporary device in these patients if they are in cardiogenic shock for resuscitation prior to a durable device to improve outcomes. At our institution, we have implanted durable and temporary VADs in 12 Fontan patients as a bridge to transplantation or destination therapy between 2018 and 2023, and this number continues to rise (Table 2). The most common diagnosis in our cohort was hypoplastic left heart syndrome (HLHS), and the most frequently used device was the HeartMate 3. Importantly, all patients underwent Fontan fenestration at the time of transplantation. Of these 12 patients, 8 (67%) have received heart transplants, 2 (17%) remain on VAD therapy, and 2 (17%) have died. The longest supported patient was over 3 years. These findings suggest promising results with VAD use in this complex patient population.

**Table 1:** Contemporary Characteristics at Time of Implant and Outcomes of Fontan VAD Support

	Bedzra et al. (N=55)	Cedars et al. (N=45)	Chen at al. (N=106)
Age (median, IQR)	10.2 [6.4−16.9]	10 (4.5−18)	10 [4.5−16.3]
Body surface area (m2)	1.2±0.5	1±0.5	1.0 [0.6−1.6]
INTERMACS Profile at Implant			
1 2 3 4−7	30% (16) 62% (33) 6% (3) 2% (1)	25% (11) 56% (24) 5% (2) 14% (6)	28% (30) 58% (61) 8% (8) 7% (7)
Device Type			
Implantable, Continuous Paracorporeal, Continuous Paracorporeal Pulsatile Percutaneous TAH	64% (35) 20% (11) 13% (7) 2% (1) 2% (1)	73% (33) 18% (8) 13% (6) 0% (0) 0% (0)	58% (61) 19% (20) 18% (19) 5% (5) 0% (0)
Device Strategy			
Bridge to Transplant, listed Bridge to Candidacy. Destination Therapy Bridge to Recovery Other	51% (28) 35% (19) 2% (1) 4% (2) 9% (5)	76% (34) 22% (10) 0% (0) 2% (1) 0% (0)	69% (73) 26% (28) 2% (2) 2% (3) 0% (0)
Duration of Support (median, IQR)	114 [18−207]	42 [18−280]	113 [43−266]
ECMO Before Implant	29% (16)	18% (8)	26% (29)
Laboratory Values			
Albumin (mg/dL) Bilirubin (mg/dL) eGFR (mL/min/1.73m2)	3.5±0.8 1.8±1.1 87.0±44.9	3.5±0.6 2.1±81.4 99.4±39.6	3.5 [3.1−4] 1.6 [0.9−2.8] 87 [58–108]
12 Month Outcomes			
Transplant On Device Recovered Mortality	56% (31) 16% (9) 6% (3) 22% (12)	70% (32) 9% (4) 0% (0) 21% (9)	53% (56) 27% (29) 2% (2) 18% (19)

Values reported as % (N), Median [IQR], or Mean ± SD as available. ECMO: Extracorporeal membrane oxygenation, eGFR: Estimated Glomerular Filtration Rate.

**Table 2:** CCHMC Fontan VAD Outcomes From 2017–2023

CCHMC VAD Outcomes	n =12
Mean age (years)	22
Female (n, %)	3 (25%)
Race White (n,%)	8 (67%)
Diagnosis	
HLHS	8 (67%)
Tricuspid Atresia	2 (17%)
DOLV	1 (8%)
DILV	1 (8%)
Weight at VAD (kg)	70 (46−72)
VAD duration (days)	109 (49−238)
Pre-VAD EDP (Median, IQR)	15 (13−17)
Pre-VAD PVRi (Median, IQR)	1.9 (1.3−2.8)
Pre-VAD Fontan pressure (Median, IQR)	17 (15−19)
INTERMACS (Mean)	2
PLE (n, %)	2 (17%)
PB (n, %)	0 (0%)
Inotropes prevad (n, %)	8 (67%)
Fontan Fenestration at time of VAD	12 (100%)
VAD to transplant duration (days)	251 (241−298)
Outcomes	
Neuro dys (n, %)	2 (17%)
Hemorrhage (n, %)	5 (42%)
AKI (n, %)	2 (17%)
MV duration (Median, IQR)	4 (1−8)
Driveline infection (n, %)	3 (25%)
Transplantation	8 (67%)
Bridge to candidacy	2 (17%)
Mortality (n, %)	2 (17%)

### Outpatient surveillance and VADs as destination therapy

The collaboration between outpatient cardiologists and advanced heart failure cardiologists is needed to reduce the risk of late implementation of advanced heart failure strategies (e.g., VAD, transplantation, etc.) and thus mortality in Fontan patients, as highlighted in a study by Lubert et al.^71^ At our institution, we have Fontan clinics where routine echocardiographic, catheterization, MRI, exercise, and other testing are obtained. Referrals for advanced therapies are not dictated solely by clinical symptoms alone, as Fontan failure can often be subclinical in nature.

Many of our patients were on VAD therapy for >1 year, one for as long as 3 years. This highlights the fact that even though VAD therapy can be used for pre-transplant optimization, some patients are choosing to live with their VAD as chronic therapy. The senior author has a congenital heart patient who has been on VAD therapy for more than 15 years. The care of these patients can only be optimized if there is significant ongoing collaboration between primary cardiologists, patients, families, Fontan clinics, heart failure/transplant specialists, and surgeons.

## EXCOR venous cannula and the fontan pump

“Biventricular” or subpulmonary and subaortic support of a Fontan patient is difficult because circuits are designed for apical cannulation of the ventricle. A new in-flow cannula, the EXCOR Venous Cannula ® by Berlin Heart ®, is an adjunct that was developed specifically to allow subpulmonary support in a single ventricle patient by redirecting systemic venous return directly into the pulmonary circulation. Its first time use in a 12-year-old patient was recently documented and was used in combination with subaortic support due to preoperative renal and liver failure.^72^ The patient was supported and rehabilitated for 2 months before being listed for transplant. Though more patients are needed to establish the efficacy of this approach, this could be used to ‘recondition’ the pulmonary circulation to pulsatile flow prior to transplantation in well-selected patients.

As previously discussed, much of Fontan failure stems from the lack of subpulmonary pump. As such, Dr. Rodefeld and colleagues have over the past two decades been developing a pump specifically designed for the complex flow of a total cavopulmonary connection that will provide low-pressure, high-volume bidirectional pulmonary blood flow augmentation. This low-energy specialized pump has allowed discussion of a new strategy whereby, at the time of Fontan operation, a subpulmonary pump is placed to function as the new ‘right heart”, thus avoiding all of the long term consequences of the Fontan Circulation.^73^ Development is ongoing.

## Future studies

Fontan patients continue to be a fast-growing, complex patient population due to great improvements in medical and surgical care since the 1970s. Because of their increased lifespan, patients often succumb to the chronic effects of Fontan circulation, necessitating transplantation. Overall, VAD therapy as a bridge to transplant for Fontan patients has promising results. Clinical and preimplantation evaluation is perhaps one of the biggest areas with room for improvement as early detection of heart failure is key to a thorough workup and subsequent optimal outcomes of medical, device or transplant therapies. Future research should also look at VAD outcomes by device and support strategy, as not all Fontan failure is equivalent, and tailored strategies could improve outcomes. There may be a place for right-sided support devices, potentially as chronic therapy to avoid issues with the Fontan circulation. Indeed, a device that could significantly improve Fontan circulation would be a next generation total artificial heart, as it could potentially resolve both subpulmonary and left ventricle issues.

## Declaration of competing interest

The authors declare the following financial interests/personal relationships which may be considered as potential competing interests: David Morales reports a relationship with Abbott Laboratories Inc that includes: consulting or advisory. David Morales reports a relationship with CorMatrix Cardiovascular Inc that includes: consulting or advisory. David Morales reports a relationship with SynCardia Systems Llc that includes: consulting or advisory. If there are other authors, they declare that they have no known competing financial interests or personal relationships that could have appeared to influence the work reported in this paper.
